# Extracellular ATP Suppresses Perlecan Core Protein Synthesis via P2Y2 Receptor-Mediated Inhibition of Akt Signaling in Cultured Vascular Endothelial Cells

**DOI:** 10.3390/ijms262210973

**Published:** 2025-11-12

**Authors:** Lihito Ikeuchi, Takato Hara, Kazuki Kitabatake, Fumiaki Uchiumi, Chika Yamamoto, Mitsutoshi Tsukimoto, Tomoya Fujie, Toshiyuki Kaji

**Affiliations:** 1 Laboratory of Environmental Health, Faculty of Pharmaceutical Sciences, Tokyo University of Science, 6-3-1 Niijuku, Katsushika 125-8585, Japan; 2 Department of Environmental Health, Faculty of Pharmaceutical Sciences, Toho University, 2-2-1 Miyama, Funabashi 274-8510, Japan; 3 Department of Radiation Biosciences, Faculty of Pharmaceutical Sciences, Tokyo University of Science, 6-3-1 Niijuku, Katsushika 125-8585, Japan; 4 Department of Gene Regulation, Faculty of Pharmaceutical Sciences, Tokyo University of Science, 6-3-1 Niijuku, Katsushika 125-8585, Japan

**Keywords:** vascular endothelial cell, perlecan, heparan sulfate proteoglycan, ATP, purinergic receptor

## Abstract

Perlecan, a major heparan sulfate proteoglycan in the vascular basement membrane, plays an essential role in maintaining endothelial barrier integrity, regulating fibroblast growth factor-2 signaling, and exerting anticoagulant activity. Although alterations in perlecan expression are implicated in the initiation and progression of atherosclerosis, the upstream regulatory mechanisms remain unclear. In this study, we investigated the effects of extracellular ATP on perlecan expression in vascular endothelial cells. ATP, but not ADP or adenosine, suppressed perlecan expression at both mRNA and protein levels in a time- and concentration-dependent manner. This suppression was recovered by knockdown of P2Y2 receptor (P2Y2R), but not by P2X4 receptor, P2X7 receptor, or P2Y1 receptor knockdown, indicating the selective involvement of P2Y2R. Mechanistically, ATP reduced Akt phosphorylation mediated by P2Y2R, and inhibition of Akt by inhibitors decreased perlecan expression, whereas inhibitors of phosphoinositide 3-kinase, mammalian target of rapamycin complex 1, extracellular signal-regulated kinase, p38 mitogen-activated protein kinase, c-Jun N-terminal kinases did not exhibit this recovery effect. These results suggest that ATP downregulates perlecan synthesis via the P2Y2R-mediated inhibition of Akt signaling. Given that ATP is markedly elevated under pathological conditions, such as inflammation and platelet activation, suppression of perlecan synthesis is an important mechanism by which ATP promotes vascular disease progression.

## 1. Introduction

Vascular endothelial cells form a monolayer that lines the luminal surface of blood vessels and plays a crucial role in maintaining vascular homeostasis by regulating the vascular tone, nutrient transport, hemostasis, and inflammatory responses [1]. Atherosclerosis is a chronic and progressive disease characterized by lipid accumulation, inflammatory cell infiltration, and fibrous tissue deposition in the arterial wall, leading to plaque formation; development of atherosclerotic lesions is initiated by vascular endothelial injury [2]. Ischemic heart disease and stroke, which are primarily caused by atherosclerosis-induced vascular stenosis, are the main contributors to cardiovascular disease-related mortality [3].

Proteoglycans consist of a core protein covalently linked to glycosaminoglycan chains, typically represented by chondroitin/dermatan sulfate and heparan sulfate proteoglycans (HSPGs) [4]. Perlecan is a large-molecular-weight HSPG that is a key component of the vascular basement membrane connecting vascular endothelial cells to the medial layer of the vessel wall [5]. It promotes vascular endothelial cell proliferation via interaction with fibroblast growth factor-2 (FGF-2) [6] and exhibits anticoagulant activity by binding to antithrombin III [7] to prevent the initiation and progression of atherosclerosis. Its expression is regulated by various stimuli in vascular endothelial cells. Notably, its expression decreases in the neointimal region during the early stages of atherosclerosis and vascular remodeling, suggesting that its downregulation plays a role in these pathological processes. We previously reported that transforming growth factor-β1 and vascular endothelial growth factor-165 induce [8,9], whereas thrombin suppresses [10] endothelial perlecan expression.

ATP is released by cells in response to physiological and mechanical stimuli and modulates cellular activity in an autocrine or paracrine manner [11]. ATP levels in the blood are typically low (≤0.1 μM) under physiological conditions [12]; however, ATP is released at high levels by vascular endothelial cells during inflammatory responses and under shear stress [13,14]. Additionally, ATP is stored at high concentrations (approximately 400 mM) in platelet-dense granules and rapidly released during hemostasis [15], suggesting that vascular endothelial cells are exposed to markedly elevated ATP levels under pathological conditions, such as inflammation and vascular injury.

Extracellular ATP exerts its effects by activating the purinergic P2 receptors, classified as ionotropic P2X and metabotropic P2Y receptors, on the plasma membrane [16]. P2X receptors function as ligand-gated ion channels allowing the influx of sodium, potassium, and calcium ions upon ATP binding, whereas P2Y receptors are G protein-coupled receptors activated by various nucleotides, depending on the subtype [17]. P2X4 receptor (P2X4R) is the predominant P2X receptor subtype that is highly permeable to calcium and mediates ATP-induced calcium influx, triggering nitric oxide (NO) production and vasodilation in vascular endothelial cells [18,19]. P2Y1 receptor (P2Y1R) and P2Y2 receptor (P2Y2R) are mainly expressed in vascular endothelial cells, and activation of P2Y receptors induces NO production, promotes cell proliferation, and enhances thrombogenicity [20,21]. Although purinergic receptor signaling is crucial for maintaining vascular homeostasis, excessive extracellular ATP promotes vascular inflammation and atherogenesis [22,23]. This suggests that extracellular ATP regulates vascular endothelial cell functions via P2 receptor activation, exerting both protective and pathological effects.

As perlecan expression in vascular endothelial cells is modulated by various extracellular stimuli, we hypothesized that elevated extracellular ATP levels alter perlecan expression levels in pathological states. In this study, we investigated the effects of extracellular ATP on perlecan expression levels in vascular endothelial cells and examined the roles of purinergic receptors and their underlying signaling pathways in mediating this process.

## 2. Results

### 2.1. Effect of Extracellular ATP on Perlecan Expression in Vascular Endothelial Cells

**Figure 1:**
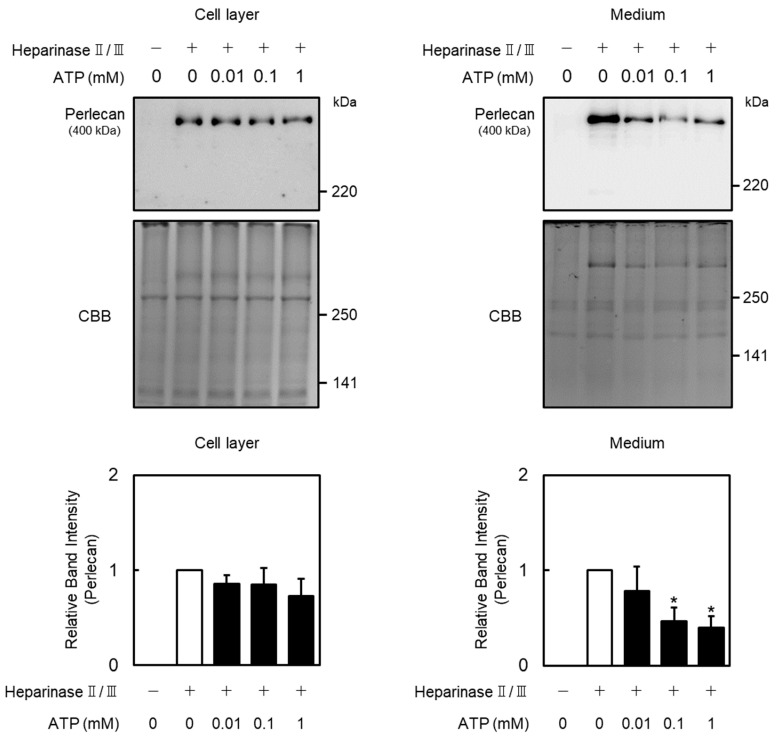
Effect of ATP on perlecan expression in vascular endothelial cells. Vascular endothelial cells were treated with or without ATP (0.01, 0.1, and 1 mM) for 12 h, perlecan core protein expression levels were determined via Western blotting. Coomassie brilliant blue (CBB) staining was used as a loading control. Each value represents the mean ± standard error (S.E.) of three independent samples. * *p* < 0.05 vs. control.

**Figure 2:**
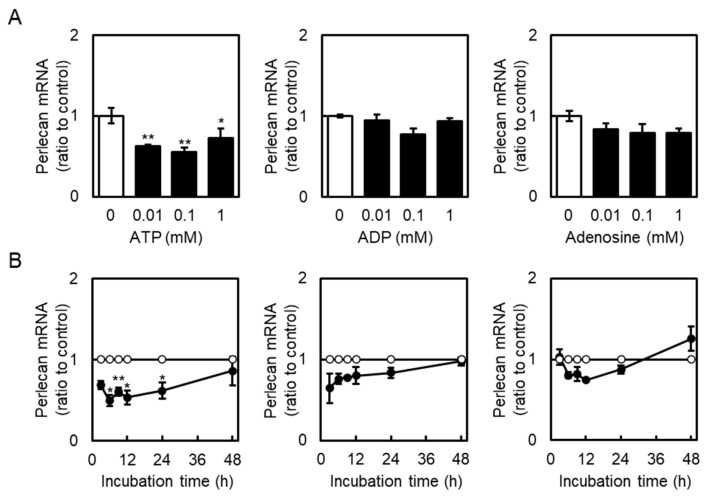
Effects of ATP, ADP, and adenosine on perlecan mRNA levels in vascular endothelial cells. (**A**) Vascular endothelial cells were treated with or without ATP, ADP, and adenosine (0.01, 0.1, and 1 mM) for 12 h, and perlecan mRNA expression levels were determined via reverse transcription polymerase chain reaction (RT-PCR). Each value represents the mean ± S.E. of three independent samples. * *p* < 0.05 and ** *p* < 0.01 vs. the corresponding untreated cells. (**B**) Vascular endothelial cells were treated with or without ATP, ADP, and adenosine (1 mM) for 3, 6, 9, 12, 24, and 48 h, and perlecan mRNA expression levels were determined via reverse transcription polymerase chain reaction (RT-PCR). Unfilled circles indicate untreated cells, whereas filled circles indicate cells treated with ATP, ADP, or adenosine. Each value represents the mean ± S.E. of three independent samples. * *p* < 0.05 and ** *p* < 0.01 vs. the corresponding untreated cells.

Effects of extracellular ATP on perlecan core protein and mRNA expression levels in vascular endothelial cells were examined. Perlecan core protein was detected at approximately 400 kDa. When vascular endothelial cells were treated with ATP, perlecan core protein levels in the cell layer did not change (Figure 1, left panel), whereas those in the conditioned medium were significantly decreased (Figure 1, right panel). Perlecan mRNA levels also decreased after treatment with ATP (0.01 mM; Figure 2A, left panel), but not ADP and adenosine (Figure 2A, middle and right panels), at 12 h. Similarly, perlecan mRNA levels initially decreased after ATP treatment at 3, 6, 12, and 24 h but subsequently recovered at 48 h (Figure 2B, left panel). No apparent cytotoxicity was observed in the cells incubated in serum-free medium for 48 h, based on morphological observation. These results suggest that ATP, but not ADP or adenosine, suppresses perlecan expression in vascular endothelial cells.

### 2.2. Involvement of P2 Receptors in the Suppression of Perlecan Expression by Extracellular ATP

**Figure 3:**
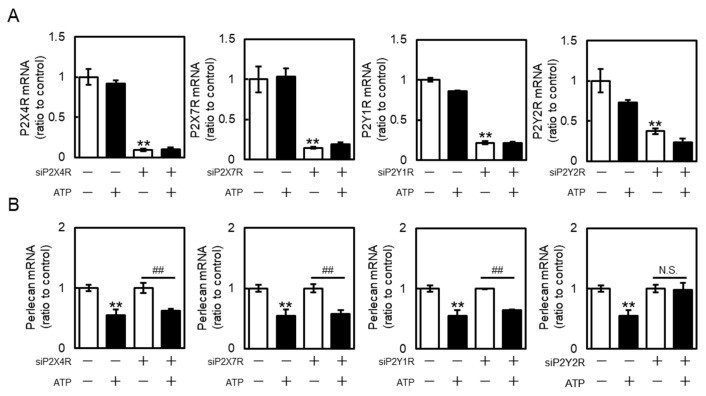
Roles of purinergic P2 receptors in perlecan suppression by ATP in vascular endothelial cells. Vascular endothelial cells transfected with the control, P2X4 receptor (P2X4R), P2X7 receptor (P2X7R), P2Y1 receptor (P2Y1R), or P2Y2 receptor (P2Y2R) small interfering RNA (siRNA) for 24 h were treated with or without ATP (1 mM) for 12 h, and (**A**) purinergic P2X4R, P2X7R, P2Y1R, P2Y2R, and (**B**) perlecan mRNA levels were determined via RT-PCR. Each value represents the mean ± S.E. of three independent samples. ** *p* < 0.01 vs. control siRNA-transfected cells without ATP; ^##^ *p* < 0.01 vs. P2X4R-, P2X7R-, P2Y1R-, or P2Y2R siRNA-transfected cells without ATP. N.S. indicates not significant.

As four types of P2 receptors (P2X4R, P2X7 receptor (P2X7R), P2Y1R, and P2Y2R) are predominantly expressed in bovine aortic endothelial cells [24], their roles in perlecan suppression by extracellular ATP were investigated. The mRNA expression of these receptors was effectively reduced by siRNA transfection (Figure 3A), consistent with our previous study, which was shown that these siRNAs also suppressed the receptor expression at the protein level [24]. P2X4R, P2X7R, or P2Y1R knockdown did not reverse the ATP-mediated suppression of perlecan in vascular endothelial cells (Figure 3B, left three panels). However, P2Y2R knockdown effectively reversed the ATP-mediated suppression of perlecan (Figure 3B, rightmost panels), suggesting that perlecan suppression by extracellular ATP is mediated by the P2Y2R in vascular endothelial cells.

### 2.3. Suppression of Endothelial Akt Signaling by Extracellular ATP

**Figure 4:**
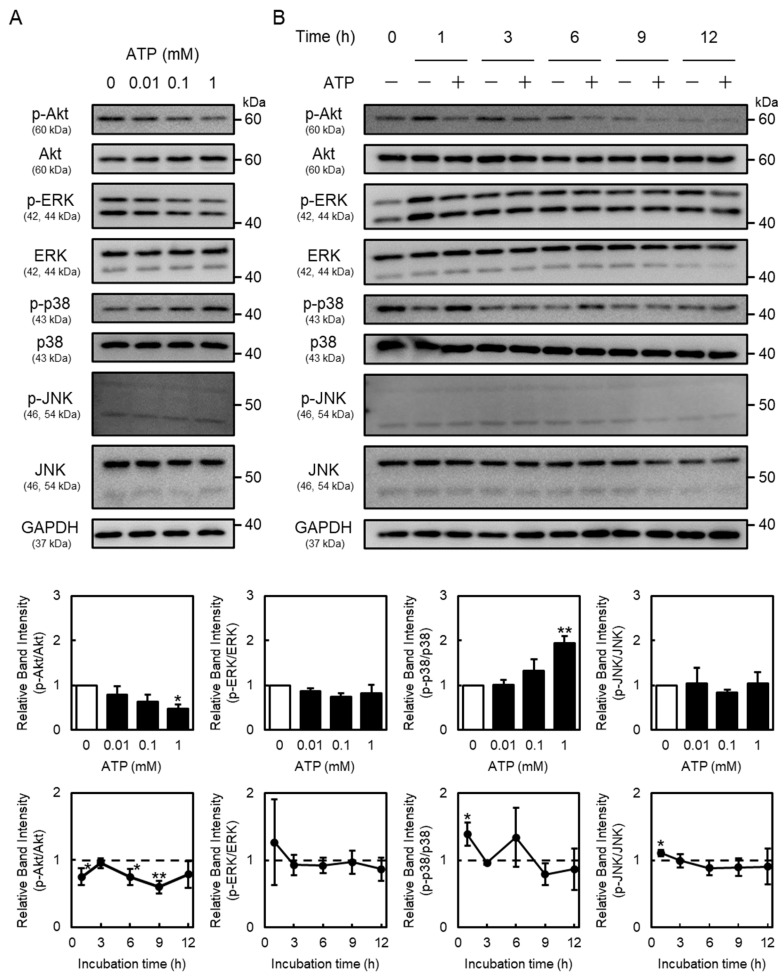
Effects of ATP on Akt and mitogen-activated protein kinase (MAPK) signaling in vascular endothelial cells. Vascular endothelial cells were treated with or without (**A**) ATP (0.01, 0.1, and 1 mM) for 1 h or (**B**) ATP (1 mM) for 1, 3, 6, 9, or 12 h, and phosphorylated Akt (p-Akt), Akt, phosphorylated extracellular signal-regulated kinase (p-ERK), ERK, phosphorylated p38 MAPK (p-p38 MAPK), p-38 MAPK, phosphorylated c-Jun N-terminal kinases (p-JNK), JNK, and glyceraldehyde 3-phosphate dehydrogenase (GAPDH) protein levels were determined via Western blotting. GAPDH was used as a loading control. Each value represents the mean ± S.E. of three independent samples. * *p* < 0.05 and ** *p* < 0.01 vs. control.

**Figure 5:**
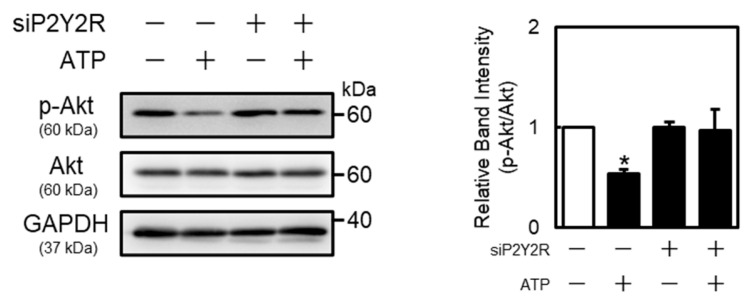
Role of P2Y2R in ATP-induced Akt phosphorylation suppression in vascular endothelial cells. Vascular endothelial cells transfected with the control or P2Y2R siRNA for 24 h were treated with or without ATP (1 mM) for 1 h, and p-Akt and Akt protein levels were determined via Western blotting. GAPDH was used as a loading control. Each value represents the mean ± S.E. of three independent samples. * *p* < 0.05 vs. control.

As the P2Y2R regulates Akt and mitogen-activated protein kinase (MAPK) (extracellular signal-regulated kinase (ERK), p38 MAPK, and c-Jun N-terminal kinases (JNK)) signaling [25,26], this study investigated the activation of Akt, ERK, p38 MAPK, and JNK by extracellular ATP in vascular endothelial cells. These proteins were detected at approximately 60 kDa (Akt), 42 and 44 kDa (ERK), 43 kDa (p38 MAPK), 46 and 54 kDa (JNK), and 36 kDa (GAPDH), respectively. Phosphorylated Akt protein levels in vascular endothelial cells were decreased by ATP in concentration- and time-dependent manners (Figure 4). Moreover, phosphorylated p38 MAPK protein levels were increased after ATP treatment at 1 h. Notably, phosphorylated JNK levels were not affected by ATP (Figure 4), suggesting that extracellular ATP suppresses the activation of only Akt signaling in vascular endothelial cells. As shown in Figure 5, phosphorylated Akt levels decreased by ATP were restored by the knockdown of P2Y2R, suggesting that Akt signaling suppression by ATP is mediated by the P2Y2R in vascular endothelial cells.

### 2.4. Roles of Phosphoinositide 3-Kinase (PI3K)/Akt and MAPK Signaling in Perlecan Expression

**Figure 6:**
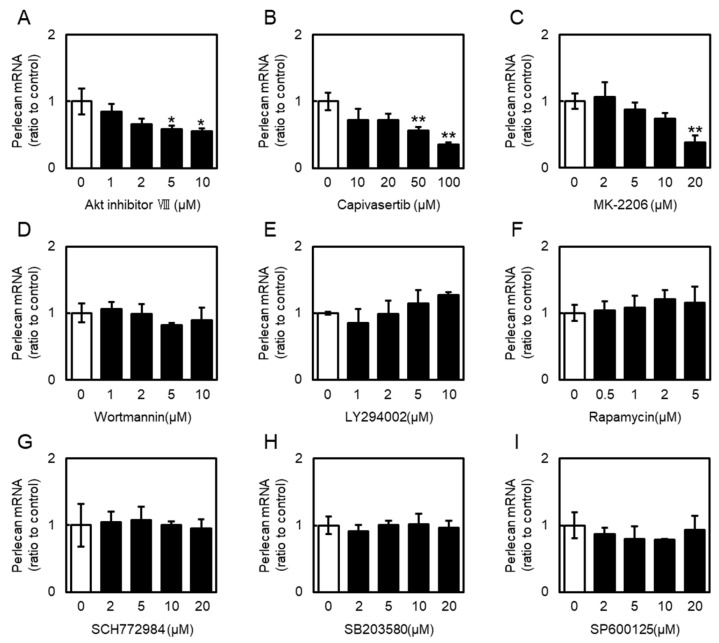
Effects of phosphoinositide 3-kinase (PI3K)/Akt pathway activators and inhibitors on perlecan mRNA levels in vascular endothelial cells. Vascular endothelial cells were treated with Inhibitors of (**A**) Akt (Akt inhibitor VIII), (**B**) Akt (capivasertib), (**C**) Akt (MK-2206), (**D**) PI3K (wortmannin), (**E**) PI3K (LY294002), (**F**) mammalian target of rapamycin complex 1 (mTORC1) (rapamycin), (**G**) ERK (SCH772984), (**H**) p38 MAPK (SB203580), and (**I**) JNK (SP600125) for 12 h. Perlecan mRNA levels were determined via RT-PCR. Each value represents the mean ± S.E. of three independent samples. * *p* < 0.05 and ** *p* < 0.01 vs. control.

Next, involvement of PI3K/Akt and MAPK signaling in perlecan expression in vascular endothelial cells was examined using specific signal inhibitors. Treatment with the three Akt inhibitors (Akt inhibitor VIII, capivasertib, and MK-2206) decreased the perlecan mRNA levels in vascular endothelial cells in a concentration-dependent manner (Figure 6A–C). Interestingly, inhibitors of PI3K (wortmannin and LY294002), mammalian target of rapamycin complex 1 (mTORC1) (rapamycin), ERK (SCH772984), p38 MAPK (SB203580), and JNK (SP600125) had no effect on perlecan mRNA levels (Figure 6D–I), suggesting that the inhibition of only Akt signaling suppresses perlecan expression in vascular endothelial cells.

## 3. Discussion

Decreased perlecan expression induces vascular endothelial detachment and enhances vascular permeability in the early phase [27], whereas loss of perlecan impairs FGF-2 signaling, promoting the abnormal proliferation and migration of vascular smooth muscle cells and intimal thickening in the later phases [28] of vascular injury. Sustained perlecan suppression reduces the anticoagulant activity and enhances thrombus formation [7,29]. Although altered perlecan synthesis is closely involved in the initiation and progression of atherosclerosis, the underlying mechanisms regulating perlecan expression remain unclear. The key findings of this study are listed below. (1) Extracellular ATP suppressed the perlecan core protein expression in vascular endothelial cells, and (2) this effect was not observed with ADP or adenosine. (3) Moreover, the suppression of perlecan expression by ATP was mediated selectively by P2Y2R, but not P2X4R, P2X7R, or P2Y1R. (4) ATP suppressed Akt phosphorylation via P2Y2R. (5) Akt inhibition decreased perlecan expression. Our results suggest that ATP suppresses perlecan expression in vascular endothelial cells via P2Y2R-mediated inhibition of Akt signaling. Uridine-5′-triphosphate (UTP), another ligand of P2Y2R, suppressed perlecan expression, indicating the selectivity of P2Y2R in regulating perlecan expression. Akt activator SC-79 failed to increase perlecan expression, suggesting that Akt signaling maintains basal perlecan synthesis but does not induce its expression. These results suggest that the local increase in ATP concentrations during vascular injury is associated with perlecan downregulation via P2Y2R–Akt signaling.

In vascular endothelial cells, perlecan maintains the endothelial barrier integrity [30,31,32] and anticoagulant activity by activating antithrombin III [7] and regulating FGF-2 signaling [6,33]. In vascular smooth muscle cells, perlecan suppresses abnormal proliferation and controls migration toward the intima via FGF-2 signaling, regulates adhesion and transmigration via negatively charged heparan sulfate chains, and modulates foam cell formation via interactions with low-density lipoproteins in monocytes and macrophages [28,34]. Therefore, reduced synthesis of perlecan in vascular endothelial cells can disrupt vascular homeostasis at multiple levels, promoting vascular endothelial dysfunction, smooth muscle cell activation, and inflammatory cell recruitment, all of which contribute to atherosclerosis progression [27,28,29]. ATP is released at high concentrations particularly from aggregated platelets under pathological conditions, such as inflammation and platelet activation [13,15,35,36], and induces vascular pathologies, including increased permeability, inflammation, and thrombosis [22,23]. Notably, ATP-induced endothelial hyperpermeability, thrombus formation, abnormal smooth muscle proliferation and migration [28], monocyte infiltration, and inflammatory activation [34] overlap with the pathological outcomes associated with perlecan suppression. This suggests that ATP-mediated vascular injury is partially mediated by perlecan downregulation in vascular endothelial cells. However, ATP also exerts perlecan-independent effects, including NO-dependent vasodilation and direct immune cell activation, and perlecan maintains growth factor storage and anticoagulant activity. Therefore, vascular injury caused by elevated ATP levels involves multiple signaling pathways, with perlecan suppression possibly serving as a key component.

P2Y1R and P2Y2R exhibit distinct ligand selectivity; P2Y1R responds primarily to ADP, whereas P2Y2R responds equally to ATP and UTP [16,37]. Although both P2Y1R and P2Y2R are G_q/11_-coupled receptors, they exhibit different downstream signaling pathways. Akt is a central serine/threonine kinase regulated by the balance between phosphorylation mediated by phosphoinositide-dependent kinase-1 (PDK1) and mammalian target of rapamycin complex 2 (mTORC2) and dephosphorylation mediated by phosphatases, such as phosphatase and tensin homolog and protein phosphatase 2A [38,39,40,41]. PDK1 is recruited to the plasma membrane via phosphatidylinositol (3,4,5)-trisphosphate (PIP3) produced by PI3K [38]. P2Y2R stimulates PI3K via phospholipase C and protein kinase C, thereby activating Akt. However, P2Y2R signaling activates protein phosphatase 2A and reduces Akt phosphorylation in Schwann cells [42]. Additionally, P2Y2R activates RhoA via integrin αVβ3, which further enhances the phosphatase and tensin homolog activity [43,44]. These reports suggest that P2Y2R both positively and negatively regulates Akt. However, inhibitory mechanisms are possibly predominant in vascular endothelial cells, leading to the ATP-induced suppression of perlecan expression. Future studies should identify the specific phosphatases involved in these mechanisms.

In conclusion, our study demonstrated that extracellular ATP suppressed perlecan synthesis via P2Y2R-mediated inhibition of Akt signaling in vascular endothelial cells. Considering the central role of perlecan in maintaining the endothelial barrier functions, regulating smooth muscle proliferation, and modulating monocyte infiltration, our findings highlight perlecan as an important mediator linking purinergic signaling to vascular homeostasis and disease progression. Although the present work focused on in vitro mechanisms, further in vivo studies using atherosclerosis models, such as *ApoE*-knockout mice, would be valuable to support this mechanism. Thus, suppression of perlecan synthesis represents an important mechanism by which elevated extracellular ATP contributes to vascular pathologies, such as atherosclerosis and thrombosis.

## 4. Materials and Methods

### 4.1. Materials

Bovine aortic endothelial cells were purchased from Cell Applications (San Diego, CA, USA). Dulbecco’s Modified Eagle Medium (DMEM) and Calcium- and magnesium-free phosphate-buffered saline (CMF-PBS) were purchased from Shimadzu Diagnostics Corporation (Tokyo, Japan). Fetal bovine serum (FBS), Lipofectamine RNAiMAX transfection reagent, Opti-MEM reduced serum medium, and MagicMark XP Western Protein Standard were purchased from Thermo Fisher Scientific (Waltham, MA, USA). Tissue culture dishes and plates were purchased from Nippone Genetics (Tokyo, Japan). ADP, ATP, and diethylaminoethyl-Sephacel were purchased from Sigma-Aldrich (St. Louis, MO, USA). Adenosine, chondroitin sulfate C sodium salt, and horseradish peroxidase-conjugated anti-glyceraldehyde-3-phosphate dehydrogenase monoclonal antibody (015-25473) were purchased from FUJIFILM Wako Pure Chemical Industries (Osaka, Japan). Inhibitors of Akt (Akt inhibitor VIII), ERK (SCH772984), p38 MAPK (SB203580), and JNK (SP600125) were purchased from Cayman Chemical (Ann Arbor, MI, USA). Capivasertib, MK-2206, and rapamycin were purchased from MedChemExpress (Monmouth Junction, NJ, USA). Wortmannin was purchased from Selleck (Houston, TX, USA). Heparinases II and III were purchased from IBEX Technologies (Montreal, QC, Canada). DynaMarker Protein MultiColor Stable II was purchased from BioDynamics Laboratory (Tokyo, Japan). Polyvinyl difluoride membrane (0.2 μm pore size) was purchased from Cytiva (Marlborough, MA, USA). Mouse monoclonal anti-perlecan antibody (sc-377219) was purchased from Santa Cruz Biotechnology (Santa Cruz, CA, USA). Rabbit monoclonal anti-phospho-Akt (Ser473; #4060), rabbit monoclonal anti-Akt (#4691), rabbit polyclonal anti-phospho-ERK1/2 (Thr202/tyr204; #9101), rabbit polyclonal anti-ERK1/2 (#9102), rabbit polyclonal anti-phospho-p38 MAPK (Thr180/Tyr182; #9211), rabbit polyclonal anti-p38 MAPK (#9212), mouse monoclonal anti-phospho-JNK (Thyr183/Thy185; #9255), rabbit polyclonal anti-JNK (#9252), horseradish peroxidase-conjugated anti-rabbit IgG (#7074), and horseradish peroxidase-conjugated anti-mouse IgG (#7076) antibodies were purchased from Cell Signaling (Beverly, MA, USA). Chemi-Lumi One Super, Coomassie brilliant blue (CBB) R-250, and other reagents were purchased from Nacalai Tesque (Kyoto, Japan).

### 4.2. Cell Culture and Treatment

Vascular endothelial cells were cultured in DMEM supplemented with 10% FBS at 37 °C in 5% carbon dioxide until confluence. The medium was removed, and the cells were washed twice with serum-free DMEM. Then, the cells were treated with or without ATP, ADP, adenosine (0.01, 0.1, and 1 mM), Akt, PI3K, mTORC1, ERK, p38 MAPK, and JNK signaling inhibitors (1, 2, 5, 10, 20, 50, and 100 µM) for 3, 6, 9, 12, 24, and 48 h in serum-free DMEM in 100 mm dishes or 12-well culture plates.

### 4.3. Small Interfering RNA (siRNA) Transfection

Next, siRNA (Fasmac, Kanagawa, Japan) transfection was performed using the Lipofectamine RNAiMAX Transfection Reagent, according to the manufacturer’s protocol. Briefly, the annealed siRNA duplex and Lipofectamine RNAiMAX were dissolved in Opti-MEM in separate tubes and incubated for 5 min at 25 °C, followed by mixing and incubation for 20 min at room temperature. Vascular endothelial cells cultured until confluence were incubated in DMEM supplemented with 10% FBS and the siRNA/Lipofectamine RNAiMAX mixture for 24 h. The final concentrations of siRNA and Lipofectamine RNAiMAX were 40 nM and 0.2%, respectively. After incubation, the cells were treated with or without ATP. The sequences of the sense and antisense strands of siRNAs were as follows: Bovine P2X4R siRNA, 5′-AUAAAUGCACGUUUUGAGGUATT-3′; bovine P2X7R siRNA, 5′-UCAUUUACCAUCCUAAUACAGTT-3′; bovine P2Y1R siRNA, 5′-AUAAUCACCAGGUAAAUGGAUTT-3′; bovine P2Y2R siRNA, 5′-UUUAUGAUGGGUUUAUGACAUTT-3′. A non-specific sequence was used as a negative control siRNA (Nippon Gene, Tokyo, Japan).

### 4.4. Reverse Transcription Polymerase Chain Reaction (RT-PCR)

Vascular endothelial cells in a 12-well culture plate were treated with or without ATP, ADP, and adenosine for 3, 6, 12, 24, and 48 h. After incubation, the conditioned medium was discarded, and the cell layer was washed twice with CMF-PBS and lysed with 200 μL of ISOGEN II (Nippon Gene). The lysate was mixed with 80 μL of deionized distilled water, followed by incubation at room temperature for 10 min. The samples were centrifuged at 12,000× *g* for 10 min at 15 °C, and the supernatant (200 µL) was harvested, mixed with the same volume of 2-propanol, and centrifuged again at 20,000× *g* for 5 min at 15 °C. Then, the supernatant was removed, and the precipitate was resuspended in 1 mL of 75% ethanol, centrifuged at 20,000× *g* for 5 min at 15 °C. The supernatant was removed, and the procedure was repeated twice. After centrifugation at 20,000× *g* for 5 min at 15 °C, the supernatant was removed, and the samples were dried before being dissolved in 10 µL of deionized distilled water. Total RNA concentration in the samples was determined using the NanoDrop Spectrophotometer (Thermo Fisher Scientific). Subsequently, cDNA was synthesized using the High-Capacity cDNA Reverse Transcription Kit (Thermo Fisher Scientific). Real-time PCR was performed using the GeneAce SYBR qPCR Mix α (Nippon Gene) with 4 ng of cDNA and primers on the StepOnePlus Real-Time PCR System (Thermo Fisher Scientific). The thermal cycling parameters were as follows: 95 °C for 10 min, followed by 40 cycles at 95 °C for 30 s and 60 °C for 1 min. Then, mRNA levels were quantified using the ΔΔCt method. The fold change in target gene expression was normalized to that of β2-microglobulin. The sequences of bovine gene-specific forward and reverse primers are listed in Table 1.

### 4.5. Perlecan Core Protein Extraction

Proteoglycans were extracted from the cell layer and conditioned medium of vascular endothelial cells under dissociative conditions as previously described [45]. Vascular endothelial cells cultured in a 100 mm culture dish were treated with or without ATP (0.01, 0.1, and 1 mM) for 12 h. After incubation, the conditioned medium was collected, and solid urea was added to a final concentration of 8 M. The cell layer was washed twice with CMF-PBS and lysed with 8 M urea cell extraction solution containing 120 mM 6-aminohexanoic acid, 12 mM benzamidine, 10 mM N-ethylmaleimide, 2 mM Ethylenediaminetetraacetic acid (EDTA), 0.1 M phenylmethanesulfonyl fluoride, 0.1 M sodium chloride, and 2% Triton X-100 in 50 mM Tris-HCl buffer (pH 7.5). The extracts were applied to the diethylaminoethyl-Sephacel (300 μL of resin) column and washed four times with 8 M urea buffer containing 0.25 M sodium chloride, 2 mM EDTA, and 0.5% Triton X-100 in 50 mM Tris-HCl buffer (pH 7.5). Proteoglycans were eluted with 0.9 mL of 8 M urea buffer containing 3 M sodium chloride, 2 mM EDTA, and 0.5% Triton X-100 in 50 mM Tris-HCl buffer (pH 7.5). The eluted samples were added to 5 μL of a 5 mg/mL chondroitin sulfate C solution and 3.5 volumes of 1.3% potassium acetate in 95% ethanol, incubated at −20 °C for 2 h, and centrifuged at 15,000× *g* for 15 min at 4 °C, and the supernatant was removed; this step was repeated until urea was completely removed. The precipitated samples were further digested with 0.02 IU/mL heparinase II/III in 100 mM Tris-HCl buffer (pH 7.0) containing 10 mM calcium acetate, 18 mM sodium acetate, 2 mM 6-aminohexanoic acid, 0.2 mM benzamidine hydrochloride monohydrate, and 20 μM phenylmethanesulfonyl fluoride at 37 °C for 3 h. The samples were lysed using sodium dodecyl sulfate sample buffer (50 mM Tris-HCl buffer solution containing 2% sodium dodecyl sulfate and 10% glycerol; pH 6.8), followed by incubation at 95 °C for 3 min. Then, Western blotting analysis was performed as described below.

### 4.6. Western Blotting Analysis

Vascular endothelial cells cultured in a 6-well culture plate were treated with or without ATP (0.01, 0.1, and 1 mM) for 1, 3, 6, 9, and 12 h. The conditioned medium was discarded, and the cell layer was washed twice with CMF-PBS and lysed using sodium dodecyl sulfate sample buffer (50 mM Tris-HCl buffer solution containing 2% sodium dodecyl sulfate and 10% glycerol; pH 6.8) and incubated at 95 °C for 15 min. Protein concentration in the samples was determined using the Protein Assay BCA kit (Nacalai Tesque). Glycerol (1.67%), 2-mercaptoethanol (1.67%), and bromophenol blue (1.67%) were added to the samples (10 µg protein) and incubated at 95 °C for 5 min. Whole cell lysates were separated via sodium dodecyl sulfate–polyacrylamide gel electrophoresis on 10% slab gels, or the extracted proteoglycan core protein lysates were separated on 4–16% gradient slab gels and electrotransferred to polyvinylidene difluoride membranes (0.2 μm pore size) at 2 mA/cm^2^ for 1 h. The membranes were blocked with 0.5% bovine serum albumin in 20 mM Tris-HCl buffer solution containing 150 mM sodium chloride and 0.1% Tween 20 (pH 7.5) for 1 h and incubated with primary antibodies (1:1000) at 4 °C overnight. After washing with 20 mM Tris-HCl buffer containing 15 mM NaCl and 0.1% Tween 20 (pH 7.5), the membranes were incubated with horseradish peroxidase-conjugated secondary antibodies (1:5000) at room temperature for 1 h. Immunoreactive bands were visualized using Chemi-Lumi One Super and scanned using the LAS 3000 Imager (FUJIFILM Wako Pure Chemical Industries). Densitometric analysis of the protein bands was performed using ImageJ software (version 1.53k) [46].

According to previous studies, CBB staining was used as a control for the perlecan core protein analysis [47,48]. The extracted perlecan core protein lysates were separated via sodium dodecyl sulfate–polyacrylamide gel electrophoresis on 4–16% gradient slab gels, and the gels were stained with the CBB staining solution containing 0.25% CBB R-250, 5% methanol, and 7.5% acetic acid for 30 min. After incubation, the staining solution was removed, and the gels were destained overnight with a destaining solution containing 25% methanol and 7.5% acetic acid. Densitometric analysis of the total CBB stained protein bands was performed using ImageJ software (version 1.53k) [46].

### 4.7. Statistical Analysis

Data were statistically analyzed via analysis of variance or Student’s *t*-test, followed by the Bonferroni/Dunn test for multiple comparisons using the Statcel software (version 4) (OMS, Tokyo, Japan). Statistical significance was set at *p* < 0.05.

## Figures and Tables

**Table 1:** The sequence of bovine gene-specific primers.

Target	Forward	Reverse
*HSPG2* (perlecan)	5′-ATGGCAGCGATGAAGCGGAC-3′	5′-TTGTGGACACGCAGCGGAAC-3′
*P2X4R*	5′-CGCCTCCTTCTGCCTGCC-3′	5′-ATGATGTCAAAGCGGATGCCA-3′
*P2X7R*	5′-CGCAGTCAATGAATACTACTACAAGAAGA-3′	5′-GGGTGAGTCGTGAAGAGACAGATG-3′
*P2Y1R*	5′-CTTCTACTACTTCAATAAGACCGACTG-3′	5′-TGCCCACCACCACGATG-3′
*P2Y2R*	5′-CTATGGCGTGGTGTGCGTG-3′	5′-GCAGTAAAGGTTGGTGTAGAAGAGG-3′
*B2M* (β2-microglobulin)	5′-CCATCCAGCGTCCTCCAAAGA-3′	5′-TTCAATCTGGGGTGGATGGAA-3′

## Data Availability

The original contributions presented in this study are included in the article. Further inquiries can be directed to the corresponding authors.

## Abbreviations

The following abbreviations are used in this manuscript:

CBB	Coomassie brilliant blue
CMF-PBS	Calcium- and magnesium-free phosphate-buffered saline
DMEM	Dulbecco’s Modified Eagle Medium
EDTA	Ethylenediaminetetraacetic acid
ERK	Extracellular signal-regulated kinase
FBS	Fetal bovine serum
FGF-2	Fibroblast growth factor-2
GAPDH	Glyceraldehyde 3-phosphate dehydrogenase
HSPGs	Heparan sulfate proteoglycans
JNK	c-Jun N-terminal kinases
MAPK	Mitogen-activated protein kinase
mTORC1	Mammalian target of rapamycin complex 1
mTORC2	Mammalian target of rapamycin complex 2
NO	Nitric oxide
P2X4R	P2X4 receptor
P2X7R	P2X7 receptor
P2Y1R	P2Y1 receptor
P2Y2R	P2Y2 receptor
PI3K	Phosphoinositide 3-kinase
RT-PCR	Reverse transcription polymerase chain reaction
S.E.	Standard error
siRNA	Small interfering RNA
UTP	Uridine-5′-triphosphate

## References

[1] Hurairah H., Ferro A. (2004). The role of the endothelium in the control of vascular function. Int. J. Clin. Pract..

[2] Ross R., Glomset J., Harker L. (1977). Response to injury and atherogenesis. Am. J. Pathol..

[3] Nedkoff L., Briffa T., Zemedikun D., Herrington S., Wright F.L. (2023). Global Trends in Atherosclerotic Cardiovascular Disease. Clin. Ther..

[4] Couchman J.R., Pataki C.A. (2012). An introduction to proteoglycans and their localization. J. Histochem. Cytochem..

[5] Melrose J. (2020). Perlecan, a modular instructive proteoglycan with diverse functional properties. Int. J. Biochem. Cell Biol..

[6] Aviezer D., Hecht D., Safran M., Eisinger M., David G., Yayon A. (1994). Perlecan, basal lamina proteoglycan, promotes basic fibroblast growth factor-receptor binding, mitogenesis, and angiogenesis. Cell.

[7] Mertens G., Cassiman J.J., Van den Berghe H., Vermylen J., David G. (1992). Cell surface heparan sulfate proteoglycans from human vascular endothelial cells. Core protein characterization and antithrombin III binding properties. J. Biol. Chem..

[8] Kaji T., Yamada A., Miyajima S., Yamamoto C., Fujiwara Y., Wight T.N., Kinsella M.G. (2000). Cell density-dependent regulation of proteoglycan synthesis by transforming growth factor-β_1_ in cultured bovine aortic endothelial cells. J. Biol. Chem..

[9] Kaji T., Yamamoto C., Oh-i M., Fujiwara Y., Yamazaki Y., Morita T., Plaas A.H., Wight T.N. (2006). The vascular endothelial growth factor VEGF165 induces perlecan synthesis via VEGF receptor-2 in cultured human brain microvascular endothelial cells. Biochim. Biophys. Acta.

[10] Fujii N., Kaji T., Akai T., Koizumi F. (1997). Thrombin reduces large heparan sulfate proteoglycan molecules in cultured vascular endothelial cell layers through inhibition of core protein synthesis. Thromb. Res..

[11] Corriden R., Insel P.A. (2010). Basal release of ATP: An autocrine-paracrine mechanism for cell regulation. Sci. Signal..

[12] Choi H.W., Ferrara K.W., Barakat A.I. (2007). Modulation of ATP/ADP concentration at the endothelial surface by shear stress: Effect of flow recirculation. Ann. Biomed. Eng..

[13] Bodin P., Burnstock G. (1998). Increased release of ATP from endothelial cells during acute inflammation. Inflamm. Res..

[14] Bodin P., Bailey D., Burnstock G. (1991). Increased flow-induced ATP release from isolated vascular endothelial cells but not smooth muscle cells. Br. J. Pharmacol..

[15] Holmsen H., Weiss H.J. (1979). Secretable storage pools in platelets. Annu. Rev. Med..

[16] North R.A. (2016). P2X receptors. Phil. Trans. R. Soc. B.

[17] von Kügelgen I., Hoffmann K. (2016). Pharmacology and structure of P2Y receptors. Neuropharmacology.

[18] Yamamoto K., Korenaga R., Kamiya A., Qi Z., Sokabe M., Ando J. (2000). P2X_4_ receptors mediate ATP-induced calcium influx in human vascular endothelial cells. Am. J. Physiol. Heart Cric. Physiol..

[19] Yamamoto K., Sokabe T., Matsumoto T., Yoshimura K., Shibata M., Ohura N., Fukuda T., Sato T., Sekine K., Kato S. (2006). Impaired flow-dependent control of vascular tone and remodeling in P2X4-deficient mice. Nat. Med..

[20] Cabou C., Martinez L.O. (2022). The Interplay of Endothelial P2Y Receptors in Cardiovascular Health: From Vascular Physiology to Pathology. Int. J. Mol. Sci..

[21] Ding L., Ma W., Littmann T., Camp R., Shen J. (2011). The P2Y_2_ nucleotide receptor mediates tissue factor expression in human coronary artery endothelial cells. J. Biol. Chem..

[22] Peikert A., König S., Suchanek D., Rofa K., Schäfer I., Dimanski D., Karnbrock L., Bulatova K., Engelmann J., Hoppe N. (2022). P2X_4_ deficiency reduces atherosclerosis and plaque inflammation in mice. Sci. Rep..

[23] Strassheim D., Verin A., Batori R., Nijmeh H., Burns N., Kovacs-Kasa A., Umapathy N.S., Kotamarthi J., Gokhale Y.S., Karoor V. (2020). P2Y Purinergic Receptors, Endothelial Dysfunction, and Cardiovascular Diseases. Int. J. Mol. Sci..

[24] Ikeuchi L., Hara T., Kitabatake K., Uchiumi F., Yamamoto C., Tsukimoto M., Fujie T., Kaji T. (2025). Expression profiles of purinergic P1 and P2 receptors in cultured bovine aortic endothelial cells, bovine aortic smooth muscle cells, and human vascular endothelial EA.hy926 cells. J. Toxicol. Sci..

[25] Ralevic V., Burnstock G. (1998). Receptors for purines and pyrimidines. Pharmacol. Rev..

[26] Gerber H.P., McMurtrey A., Kowalski J., Yan M., Keyt B.A., Dixit V., Ferrara N. (1998). Vascular endothelial growth factor regulates endothelial cell survival through the phosphatidylinositol 3′-kinase/Akt signal transduction pathway. Requirement for Flk-1/KDR activation. J. Biol. Chem..

[27] Gustafsson E., Almonte-Becerril M., Bloch W., Costell M. (2013). Perlecan maintains microvessel integrity in vivo and modulates their formation in vitro. PLoS ONE.

[28] Tran P.K., Tran-Lundmark K., Soininen R., Tryggvason K., Thyberg J., Hedin U. (2004). Increased intimal hyperplasia and smooth muscle cell proliferation in transgenic mice with heparan sulfate-deficient perlecan. Circ. Res..

[29] Nugent M.A., Nugent H.M., Iozzo R.V., Sanchack K., Edelman E.R. (2000). Perlecan is required to inhibit thrombosis after deep vascular injury and contributes to endothelial cell-mediated inhibition of intimal hyperplasia. Proc. Natl. Acad. Sci. USA.

[30] Brown J.C., Sasaki T., Göhring W., Yamada Y., Timpl R. (1997). The C-terminal domain V of perlecan promotes β1 integrin-mediated cell adhesion, binds heparin, nidogen and fibulin-2 and can be modified by glycosaminoglycans. Eur. J. Biochem..

[31] Hayashi K., Madri J.A., Yurchenco P.D. (1992). Endothelial cells interact with the core protein of basement membrane perlecan through beta 1 and beta 3 integrins: An adhesion modulated by glycosaminoglycan. J. Cell Biol..

[32] Woodley D.T., Rao C.N., Hassell J.R., Liotta L.A., Martin G.R., Kleinman H.K. (1983). Interactions of basement membrane components. Biochim. Biophys. Acta.

[33] Whitelock J.M., Murdoch A.D., Iozzo R.V., Underwood P.A. (1996). The degradation of human endothelial cell-derived perlecan and release of bound basic fibroblast growth factor by stromelysin, collagenase, plasmin, and heparanases. J. Biol. Chem..

[34] Evanko S.P., Raines E.W., Ross R., Gold L.I., Wight T.N. (1998). Proteoglycan distribution in lesions of atherosclerosis depends on lesion severity, structural characteristics, and the proximity of platelet-derived growth factor and transforming growth factor-beta. Am. J. Pathol..

[35] Burnstock G., Knight G.E. (2004). Cellular distribution and functions of P2 receptor subtypes in different systems. Int. Rev. Cytol..

[36] Yegutkin G.G. (2008). Nucleotide- and nucleoside-converting ectoenzymes: Important modulators of purinergic signalling cascade. Biochim. Biophys. Acta.

[37] von Kügelgen I. (2006). Pharmacological profiles of cloned mammalian P2Y-receptor subtypes. Pharmacol. Ther..

[38] Alessi D.R., James S.R., Downes C.P., Holmes A.B., Gaffney P.R., Reese C.B., Cohen P. (1997). Characterization of a 3-phosphoinositide-dependent protein kinase which phosphorylates and activates protein kinase Balpha. Curr. Biol..

[39] Sarbassov D.D., Guertin D.A., Ali S.M., Sabatini D.M. (2005). Phosphorylation and regulation of Akt/PKB by the rictor-mTOR complex. Science.

[40] Campbell R.B., Liu F., Ross A.H. (2003). Allosteric activation of PTEN phosphatase by phosphatidylinositol 4,5-bisphosphate. J. Biol. Chem..

[41] Maehama T., Dixon J.E. (1998). The tumor suppressor, PTEN/MMAC1, dephosphorylates the lipid second messenger, phosphatidylinositol 3,4,5-trisphosphate. J. Biol. Chem..

[42] Coover R.A., Healy T.E., Guo L., Chaney K.E., Hennigan R.F., Thomson C.S., Aschbacher-Smith L.E., Jankowski M.P., Ratner N. (2018). Tonic ATP-mediated growth suppression in peripheral nerve glia requires arrestin-PP2 and is evaded in NF1. Acta Neuropathol. Commun..

[43] Erb L., Liu J., Ockerhausen J., Kong Q., Garrad R.C., Griffin K., Neal C., Krugh B., Santiago-Pérez L.I., González F.A. (2001). An RGD sequence in the P2y_2_ receptor interacts with α_V_β_3_ integrins and is required for G_o_-mediated signal transduction. J. Cell Biol..

[44] Hsu Y.H., Chang C.C., Yang N.J., Lee Y.H., Juan S.H. (2014). RhoA-mediated inhibition of vascular endothelial cell mobility: Positive feedback through reduced cytosolic p21 and p27. J. Cell. Physiol..

[45] Hara T., Kojima T., Matsuzaki H., Nakamura T., Yoshida E., Fujiwara Y., Yamamoto C., Saito S., Kaji T. (2017). Induction of Syndecan-4 by Organic-Inorganic Hybrid Molecules with a 1,10-Phenanthroline Structure in Cultured Vascular Endothelial Cells. Int. J. Mol. Sci..

[46] Schneider C.A., Rasband W.S., Eliceiri K.W. (2012). NIH Image to ImageJ: 25 years of image analysis. Nat. Met..

[47] Jungmann O., Nikolovska K., Stock C., Schulz J.N., Eckes B., Riethmüller C., Owens R.T., Iozzo R.V., Seidler D.G. (2012). The dermatan sulfate proteoglycan decorin modulates α2β1 integrin and the vimentin intermediate filament system during collagen synthesis. PLoS ONE.

[48] Empere M., Wang X., Prein C., Aspberg A., Moser M., Oohashi T., Clausen-Schaumann H., Aszodi A., Alberton P. (2023). Aggrecan governs intervertebral discs development by providing critical mechanical cues of the extracellular matrix. Front. Bioeng. Biotechnol..

